# Oxyphenisatin acetate (NSC 59687) triggers a cell starvation response leading to autophagy, mitochondrial dysfunction, and autocrine TNFα-mediated apoptosis

**DOI:** 10.1002/cam4.107

**Published:** 2013-07-23

**Authors:** Bethanie L Morrison, Michael E Mullendore, Luke H Stockwin, Suzanne Borgel, Melinda G Hollingshead, Dianne L Newton

**Affiliations:** 1 Drug Mechanism Group, Biological Testing Branch, Developmental Therapeutics Program, SAIC-Frederick Inc., Frederick National Laboratory for Cancer ResearchFrederick, Maryland, 21702; 2 In Vivo Preclinical Support Group, Biological Testing Branch, Developmental Therapeutics Program, SAIC-Frederick Inc., Frederick National Laboratory for Cancer ResearchFrederick, Maryland, 21702; 3 Biological Testing Branch, Developmental Therapeutics Program, Division of Cancer Treatment and Diagnosis, Frederick National Laboratory for Cancer ResearchFrederick, Maryland, 21702

**Keywords:** Autophagy, breast cancer, oxyphenisatin, protein synthesis, TNFα

## Abstract

Oxyphenisatin (3,3-bis(4-hydroxyphenyl)-1H-indol-2-one) and several structurally related molecules have been shown to have in vitro and in vivo antiproliferative activity. This study aims to confirm and extend mechanistic studies by focusing on oxyphenisatin acetate (OXY, NSC 59687), the pro-drug of oxyphenisatin. Results confirm that OXY inhibits the growth of the breast cancer cell lines MCF7, T47D, HS578T, and MDA-MB-468. This effect is associated with selective inhibition of translation accompanied by rapid phosphorylation of the nutrient sensing eukaryotic translation initiation factor 2α (eIF2α) kinases, GCN2 and PERK. This effect was paralleled by activation of AMP-activated protein kinase (AMPK) combined with reduced phosphorylation of the mammalian target of rapamycin (mTOR) substrates p70S6K and 4E-BP1. Microarray analysis highlighted activation of pathways involved in apoptosis induction, autophagy, RNA/protein metabolism, starvation responses, and solute transport. Pathway inhibitor combination studies suggested a role for AMPK/mTOR signaling, de novo transcription and translation, reactive oxygen species (ROS)/glutathione metabolism, calcium homeostasis and plasma membrane Na^+^/K^+^/Ca^2+^ transport in activity. Further examination confirmed that OXY treatment was associated with autophagy, mitochondrial dysfunction, and ROS generation. Additionally, treatment was associated with activation of both intrinsic and extrinsic apoptotic pathways. In the estrogen receptor (ER) positive MCF7 and T47D cells, OXY induced TNFα expression and TNFR1 degradation, indicating autocrine receptor-mediated apoptosis in these lines. Lastly, in an MCF7 xenograft model, OXY delivered intraperitoneally inhibited tumor growth, accompanied by phosphorylation of eIF2α and degradation of TNFR1. These data suggest that OXY induces a multifaceted cell starvation response, which ultimately induces programmed cell death.

The mechanistic basis for oxyphenisatin acetate anti-cancer activity remains unresolved. This study demonstrates that exposure is associated with an acute nutrient deprivation response leading to translation inhibition, induction of autophagy, transient estrogen receptor (ER) stress and mitochondrial dysfunction. Ultimately these effects promote apoptosis induction, which in ER+ breast cancer cells is mediated by autocrine TNFα production. This is the first study implicating a nutrient deprivation response as central to the downstream effects of oxyphenisatin acetate.

## Introduction

Considerable success has been achieved through repurposing clinically approved agents for anticancer indications 1. For example, the histone deacetylase (HDAC) inhibitor valproic acid 2, used to treat psychiatric conditions, also forces differentiation of cancer cells 3 and is currently the focus of clinical evaluation 4,5. Similarly, the classical immunosuppressive rapamycin, an inhibitor of mammalian target of rapamycin (mTOR), impacts tumor growth and has been approved for the treatment of several malignancies 6. These precedents highlight the value of mining the existing pharmacopeia for growth inhibitory activity.

Oxyphenisatin acetate (OXY) (NSC 59687), is a diphenyl oxindole originally used as a laxative in the 1950s and 1960s 7. As a stimulant laxative, OXY is recognized to alter membrane permeability leading to electrolyte flux and net loss of water 8,9. Subsequently, OXY and structurally similar diphenolics (La-96 and bisacodyl) were shown to inhibit the growth of transformed cells in vitro 9. More recently, OXY was identified as having a similar spectrum of activity to CCI-779, an inhibitor of mTOR 10,11. Analogs of OXY generated in the same study inhibited translation, leading to growth inhibition and ultimately caspase-dependent apoptosis 12. These effects were associated with rapid activation of the eukaryotic translation initiation factor 2α (eIF2α) kinase GCN2, a sensor of uncharged tRNA 13. Inhibition of mTOR signaling was also inferred through reduced phosphorylation of p70S6k. Encouragingly, antitumor activity was also observed in mouse xenograft models. These observations strongly support the idea that the oxyphenisatin pharmacophore is a metabolic modulator. However, a unifying mechanism for this class of agent remains undefined.

In this study, to begin the search for a molecular target, we sought to confirm and extend mechanism studies for this class of molecule by focusing on the oxyphenisatin analog, OXY. We chose to study OXY because data from the NCI60 cell line screen showed this analog to have a relatively narrow spectrum of activity; that is, OXY was mainly cytotoxic toward breast and ovarian cancer cell lines. Our results validated previous reports with respect to growth arrest with inhibition of translation. Likewise, we confirmed activation of the eIF2α kinase GCN2 and also showed transient activation of another eIF2α kinase, PERK (protein kinase-like endoplasmic reticulum kinase), a sensor of estrogen receptor (ER) stress 13. Concurrent phosphorylation of the ATP sensor, AMP-activated protein kinase (AMPK), was also noted. Novel data from cDNA microarray analysis and pathway inhibitor assays implicated several signaling pathways in OXY activity. For example, experiments showed induction of autophagy and mitochondrial dysfunction. Furthermore, OXY was shown to induce apoptosis through stimulation of an autocrine TNFα (tumor necrosis factor alpha) pathway in estrogen receptor positive (ER^+^) cell lines. These data support the hypothesis that nutrient depletion at the level of amino acids and ATP drives several facets of the response to OXY, leading ultimately to apoptosis induction. Collectively, these observations confirm the unique mechanism of OXY and refine the list of molecular targets responsible for activity.

## Materials and Methods

### Materials

OXY, NSC 59687 and oxyphenisatin (NSC 59814) were obtained from the Drug Synthesis and Chemistry Branch of the Developmental Therapeutics Program, National Cancer Institute (Rockville, MD). All cell lines were from the Division of Cancer Treatment and Diagnosis Tumor Repository (Frederick, MD). The identity of all cell lines used in this study was confirmed using Identifiler STR genotyping (Applied Biosystems, Foster, CA). Unless otherwise stated, all other reagents and inhibitors were from Sigma (St. Louis, MO).

### Cell viability and proliferation assays

[^14^C]-leucine cell viability assays were performed as described earlier 14. Proliferation was determined with the CellTiter96 Assay (Promega, Madison, WI) following manufacturer's instructions.

### RNA–DNA–protein synthesis assay

Cells were seeded into a 96-well plate 24 h prior to treatment and the assay conducted as described 15.

### Determination of ATP and AMP levels

ATP and AMP were extracted from cells following the protocol described in Decosterd et al. 16. After treatment with OXY for the times noted, cells were trypsinized and washed three times with ice-cold PBS (phosphate buffered saline) before the addition of equal volumes of ice-cold H_2_O (10 × 10^6^ cells/100 μL) and 6% TCA (trichloroacetic acid). The cell extracts were centrifuged (4°C top speed microfuge) for 20 min before analysis (50 μL) on a Whatman Partisil 10 SAX (4.6 × 250 mm) column (GE Healthcare Sciences, Piscataway, NJ). Chromatography was performed in isocratic mode (0.3 mol/L KH_2_PO_4_, pH 3.0) with a variable flow rate of 0.5 mL/min for 20 min followed by 2.0 mL/min for the last 40 min (modification of Arezzo et al. 17). AMP and ATP at 254 nm were identified by comparing the retention times of standard AMP or ATP with peaks found in the lysate, and by spiking varying amounts of standard AMP or ATP into the lysates. Experiments were performed twice with triplicate determinations for each time point and the data pooled.

### Western blotting

Western blotting was performed as described in Glaros et al. 18. Cells were harvested in SDS-loading buffer (160 mmol/L Tris-HCl (pH 6.8), 20% glycerol, 4% SDS, 4% β-mercaptoethanol), sonicated, and the insoluble material removed by centrifugation for 20 min at 11,000 × g. Resulting protein was equally loaded after adjustment based on the Coomassie stained SDS-PAGE (sodiumdodecyl sulfate polyacrylamide gel electrophoresis) gel. Following transfer to either a polyvinylidene fluoride (PVDF) or nitrocellulose membrane, membranes were blocked in 1.5% Blotto for 1 h and incubated with primary antibody overnight at 4°C. The next day, the membranes were incubated for 1 h at room temperature before being washed in TBST (Tris buffered saline + tween 20) and incubated with the horseradish peroxidase (HRP)-conjugated secondary antibody 1 h at room temperature (Molecular Probes, Carlsbad, CA). Following multiple washes in TBST, bands were visualized using Visualizer Western Blotting Detection Kit (Millipore, Billerica, MA) according to the manufacturer's protocol. Immunoblots were scanned using a Kodak Image Station 4000 Pro and captured using Kodak Molecular Imaging software (Carestream Health, New Haven, CT). All primary antibodies used were obtained from Cell Signaling Technologies (Danvers, MA) except the following: anti-p-GCN2 (Abcam, Cambridge, MA) and anti-β-actin (Sigma).

### ROS detection by flow cytometry

CM-H_2_-DCFDA (Life Technologies, Carlsbad, CA) at 10 μmol/L was added to a 1 × 10^6^/mL cell suspension for 2 h followed by 1 h of drug treatment. H_2_O_2_ (0.1%) was used as a positive control for peroxide generation. Reactive oxygen species (ROS) generation was measured as the increase in green fluorescence intensity using the FL1 channel on a FACSCaliber flow cytometer (Becton Dickinson, Franklin Lakes, NJ).

### Immunocytochemistry

Cells seeded on coverslips 24 h prior to treatments were washed twice with PBS and fixed for 15 min in 4% paraformaldehyde. They were then washed three times (5 min each) followed by 10 min incubation in 0.5% Triton X-100 for permeabilization. Following another set of washes, the coverslips were blocked in 4% BSA for at least 1 h at room temperature, primary antibodies added at a dilution of 1:250 in 4% BSA, and incubation continued overnight at 4°C. The following day, coverslips were again washed three times in PBS, incubated for 30 min at room temperature in the dark with AlexaFluor-488-conjugated secondary antibodies and rhodamine phalloidin where indicated. Following washing and mounting onto glass slides using the ProLong Gold antifade reagent containing DAPI (Molecular Probes, Carlsbad, CA), cells were visualized using a Leica DM compound microscope and BioQuant Image Analysis software (Bioquant Image Analysis Corp., Nashville, TN). Primary antibodies used included anticytochrome c (BD Biosciences, San Jose, CA), anti-Bax, LC3B (Cell Signaling Technologies, Danvers, MA), and anti-MTCO2 (Abcam, Cambridge, MA).

### Acridine orange staining

Cells were seeded onto coverslips and treated with OXY as indicated. Acridine orange (1 μg/mL in serum-free media) was added for 15 min prior to gentle washing with PBS. Coverslips were mounted onto glass slides with the ProLong Gold antifade reagent, dried, and visualized as noted above.

### Mitochondrial membrane depolarization assays

MCF7 cells on coverslips were treated for 24 h prior to the addition of JC-1 stain (2 μmol/L, Molecular Probes, Carlsbad, CA) for 30 min at 37°C. Coverslips were washed gently in PBS and immediately dry mounted to glass slides and viewed as noted above.

### Transfection of siRNA

MCF7 cells (2.5 × 10^5^) were plated into each well of a 6-well plate. Cells were transfected 24 h later with 100 nmol/L siGENOME NonTargeting siRNA Pool #1 or siGENOME SMARTpool siRNA (Dharmacon, Thermo Fisher, Lafayette, IL) directed at TNFR1 using Lipofectamine 2000 according to the manufacturer¹s instructions (Life Technologies, Carlsbad, CA). After incubation for 5 h, cells were washed once with PBS and grown in standard growth media overnight. Drug treatment was performed 24 h later.

### Microarray analysis

Total RNA was isolated from MCF7 cells treated with 10 μmol/L OXY for 24 h and the microarray procedure performed as described previously using the GeneChip Human U133 plus 2.0 array 19. Pairwise analysis was performed on control versus treated arrays using a fivefold change cutoff, <0.01 adjusted *P*-value, GC-RMA normalization with Benjamini–Hochberg false discovery estimation.

### Real-time PCR

Total RNA was isolated from cells treated with 10 μmol/L OXY for 24 h in triplicate with the RNeasy Mini Kit (Qiagen, Valencia, CA) per manufacturer's instructions, and quantified with a Nanodrop spectrophotometer system. Equal amounts of RNA were digested with DNase to remove any residual genomic DNA, followed by cDNA generation with Oligo-dT and the Superscript III first-strand synthesis system (Invitrogen, Carlsbad, CA). Quantitative real time polymerase chain reaction (qRT-PCR) was performed on an ABI-7500 to look at the expression of the following genes using Sybr-Green: GUSB (housekeeping), TWEAK (TNF-related weak inducer of apoptosis), TRAIL (TNF-related apoptosis-inducing ligand), FASL (Fas ligand), and TNFα. Primer sequences and PCR conditions are available upon request. Real-time PCR data was analyzed using the 2−ΔΔCT method.

### MCF7 xenograft model

In vivo antitumor activity was assessed using standard methodologies 20. Subcutaneous xenografts were established in 6-week-old female athymic nude mice (NCI Animal Production Program, Frederick, MD) by trocar implantation of MCF-7 tumor fragments collected from donor mice previously implanted with MCF-7 cells. As MCF-7 is an estradiol dependent tumor, its growth was supported by once weekly subcutaneous administration of 1 mg estradiol cypionate/kg of body weight. When tumors reached ∼120 mg, mice were randomized into treatment groups and therapy was initiated. Initially, a simple toxicity assessment to determine tolerability to OXY was conducted by administering single intraperitoneal (IP) doses of compound at 100, 200, and 400 mg/kg. The mice were observed for adverse effects for 14 days postdose. No toxicity was noted at any of the three dose levels administered. Assessment in several other tumor models demonstrated tolerability with OXY at 300 mg/kg given once daily or 200 mg/kg given twice daily. For the MCF-7 study treatments were administered on an exact body weight basis using dose volumes of 1–2 mL/kg body weight. The vehicle control (*n* = 18) received 100% DMSO (dimethyl sulfoxide) the treated group (*n* = 8) 300 mg OXY/kg once daily for a total of 10 days (QD × 10), followed by a 3 day rest and an additional 6 days of dosing. The dose solutions were prepared in 100% DMSO, aliquoted and stored frozen until used. The mice were monitored for a total of 52 days with treatment initiation occurring on day 27 posttumor implantation. Tumor growth was monitored by caliper measurements, and tumor weights were calculated as: (tumor length in mm × [tumor width^2^ in mm])/2 = weight in mg 20. Differences in tumor weights between the treated and control groups were assessed by Student's *t*-test using Microsoft Excel. *P* values of 0.05 or less were regarded as significant. Toxicity was monitored by clinical observations and body weight monitoring.

## Results

### OXY (NSC 59687) in vitro activity

**Figure 1:**
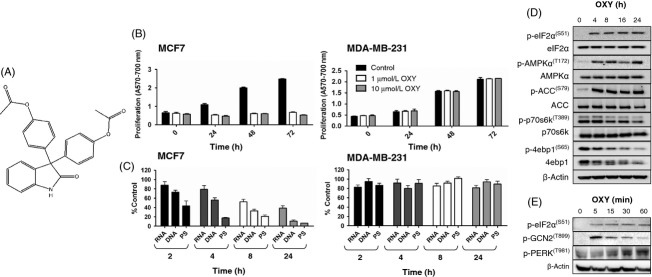
Oxyphenisatin acetate (OXY) in vitro activity. (A) Structure of NSC59687, OXY. (B) MTT assay on MCF7 or MDA-MB-231 cells after treatment with 1 μmol/L or 10 μmol/L OXY for the times indicated. The results are reported as the mean + SD of four determinations. (C) MCF7 and MDA-MB-231 cells were treated with 10 μmol/L OXY and RNA, DNA, or protein synthesis determined using [^3^H] uridine, [^3^H] thymidine, or [^14^C] leucine incorporation, respectively. Assays were performed at least twice with triplicate determinations for each point and the data pooled. Results are expressed as % control IC_50_. (D) MCF7 cells were treated with 10 μmol/L OXY for the times indicated and western blotted for expression of translation-related proteins, mammalian target of rapamycin (mTOR) and AMP-activated protein kinase (AMPK) pathway components along with (E) corresponding phosphostates of eukaryotic translation initiation factor 2α (eIF2α) kinases.

This study focused on the effects of OXY in breast cancer cell lines given that NCI 60 cell line screening data suggested that a subset of these lines was especially sensitive (data available at http://dtp.nci.nih.gov/docs/dtp_search.html). The antiproliferative activity of OXY (structure Fig. 1A) was first confirmed using a [^14^C] leucine viability assay. Results demonstrated that following treatment for 24 h, MCF7 and T47D (IC_50_ 0.8 and 0.6 μmol/L, respectively) were more sensitive than MDA-MB-468 and HS578T (IC_50_ 1.8 and 2.1 μmol/L, respectively), while MDA-MB-231 cells were resistant (IC_50_ >100 μmol/L). Results from an MTT (dimethyl thiazolyl diphenyl tetrazolium salt) assay over 72 h suggested that OXY activity was associated with growth arrest in MCF7 cells, but in resistant MDA-MB-231, cell growth continued unabated (Fig. 1B). In addition, cell cycle analysis (results not shown) appeared to suggest a slight G_2_/M arrest in sensitive cell lines following treatment.

### Effects on translation

Analogs of OXY have also been reported to inhibit translation through eIF2α and AMPK 12. Phosphorylation of eIF2α (inhibitory) occurs under conditions of amino acid deprivation, whereas AMPK is phosphorylated (activated) when intracellular ATP declines. Results from RNA, DNA, and protein synthesis assays (Fig. 1C) confirmed that OXY preferentially inhibited protein synthesis in MCF7 cells whereas in MDA-MB-231 cells no effect was observed. Western blotting (Fig. 1D) confirmed rapid phosphorylation of eIF2α and also AMPKα – along with one of its substrates acetyl CoA carboxylase (ACC). An inhibitory effect was also noted for the mTOR substrates p70S6K and 4E-BP1. Subsequent experiments (Fig. 1E) identified activation of GCN2 (sensor of uncharged tRNA) and PERK (sensor of ER stress) as candidate upstream kinases responsible for eIF2α activation. A further eIF2α kinase PKR was not activated. Examination of a panel of breast cancer cell lines confirmed rapid activation of eIF2α, GCN2, and AMPKα in sensitive lines following treatment (data not shown). Overall, these results confirm OXY acts to inhibit protein synthesis with rapid activation of eIF2α kinases GCN2 and PERK. This observation is accompanied by a phosphorylation of AMPK, which is suggestive of reduced cytosolic ATP.

### Microarray analysis

To provide an unbiased entry point for further mechanistic studies, microarray analysis was then performed on OXY-treated MCF7 cells. cDNA from 24 h treated/control cells was hybridized to duplicate Human U133 Plus 2.0 cDNA arrays. Pairwise analysis was performed using a fold change cutoff of >5 and an adjusted *P* value of <0.01. Differential regulation was noted for 790 transcripts (232 downregulated, 558 upregulated). Transcripts showing the highest upregulation included the tumor suppressor PTEN (phosphatase and tensin homolog) (74.1-fold), the death ligand receptor FAS (tumor necrosis factor receptor superfamily member 6) (66.6-fold), and GADD45A (38.7-fold). The complete list of differentially regulated transcripts is shown in Table S1. Upregulated genes were then subjected to gene ontology analysis and subdivided according to biological pathways. Results demonstrated activation of pathways involved in apoptosis induction, growth arrest, autophagy, RNA/protein metabolism, cellular starvation responses, and solute transport (Table 1).

**Table:** Microarray analysis of MCF7 cells treated with 10 μmol/L oxyphenisatin acetate (OXY)

Category	Fold change	Abbreviation	Gene ID	Name
Apoptosis	66.63	FAS	Z70519	TNF receptor superfamily, member 6
	18.23	STK17B	NM_004226	Serine/threonine kinase 17b
	17.29	RASSF1	NM_007182	Ras association (RalGDS/AF-6) domain family member 1
	16.38	PHLDA3	NM_012396	Pleckstrin homology-like domain, family A, member 3
	14.61	ATF3	NM_001674	Activating transcription factor 3
	12.37	TP53INP1	AW341649	Tumor protein p53 inducible nuclear protein 1
	11.89	BNIPL	W69365	BCL2/adenovirus E1B 19kD interacting protein like
	11.26	AEN	NM_022767	Apoptosis enhancing nuclease
	10.96	TCF25	AK024679	Transcription factor 25 (basic helix–loop–helix)
	9.35	BCL2L11	NM_006538	BCL2-like11 (apoptosis facilitator)
	8.92	MUDENG	BC013174	MU-2/AP1M2 domain containing, death-inducing
	8.82	CDKN2AIP	NM_017632	CDKN2A interacting protein
	8.22	PPP1R15A	NM_014330	Protein phosphatase 1, regulatory (inhibitor) subunit 15A
	7.61	TNFRSF10B	AF153687	Tumor necrosis factor receptor superfamily, member 10b
	6.27	TNFAIP3	AI738896	Tumor necrosis factor, alpha-induced protein 3
	6.24	ARHGEF7	AI040887	Rho guanine nucleotide exchange factor (GEF) 7
	6.12	TNFRSF10A	W65310	Tumor necrosis factor receptor superfamily, member 10a
	5.85	MAP3K9	AF251442	Mitogen-activated protein kinase kinase kinase 9
	5.47	TNFRSF12A	NM_016639	Tumor necrosis factor receptor superfamily, member 12A
Growth arrest	38.71	GADD45A	NM_001924	Growth arrest and DNA-damage-inducible, alpha
	25.04	BTG2	NM_006763	BTG family, member 2
	24.16	MDM2	AF201370	Mdm2 p53 binding protein homolog (mouse)
	18.96	SMEK2	BC032531	SMEK homolog 2, suppressor of mekl (Dictyostelium)
	18.72	POLH	AW665155	Polymerase (DNAdirected), eta
	18.4	LATS2	AI745254	LATS, large tumor suppressor, homolog 2 (Drosophila)
	17.29	RASSF1	NM_007182	Ras association (RalGDS/AF-6) domain family member 1
	15.74	PP2R5C	AW772123	Protein phosphatase 2, regulatory subunit B', gamma isoform
	15.1	CDKN1A	NM_000389	Cyclin-dependent kinase inhibitor 1A (p21, Cip1)
	14.61	ATF3	NM_001674	Activating transcription factor 3
	14.2	TERF2	BC024890	Telomeric repeat binding factor 2
	13.81	MXD1	AW071793	MAX dimerization protein 1
	13.2	FANCD2	BC013582	Fanconi anemia, complementation group D2
	13.05	ZMAT3	NM_022470	Zinc finger, matrin type 3
	11.26	AEN	NM_022767	Apoptosis enhancing nuclease
	10.12	FHL2	NM_001450	Four and a half LIM domains 2
	8.79	SKIL	NM_005414	SKI-like oncogene
	8.68	DDB2	BF970185	Damage-specific DNA binding protein 2
	8.6	BUB1	AU156551	Budding uninhibited by benzimidazoles 1 homolog (yeast)
	8.22	PPP1R15A	NM_014330	Protein phosphatase 1, regulatory (inhibitor) subunit 15A
	7.94	SESN1	NM_014454	Sestrin 1
Autophagy	15.56	WDFY3	AI732512	WD repeat and FYVE domain containing 3
	11.89	BNIPL	W69365	BCL2/adenovirus E1B 19kD interacting protein like
	11.03	ATG7	BE048026	ATG7 autophagy related 7 homolog (S. cerevisiae)
	9.24	NEU1	U84246	Sialidase 1 (lysosomal sialidase)
	8.82	CDKN2AIP	NM_017632	CDKN2A interacting protein
	8.79	SKIL	NM_005414	SKI-like oncogene
RNA/protein degradation, synthesis, transport	21.96	MPHOSPH6	BC029395	M-phase phosphoprotein 6
	20.69	CUL1	AI628926	Cullin 1
	15.56	WDFY3	AI732512	WD repeat and FYVE domain containing 3
	14.18	UBA6	BC031637	Ubiquitin-like modifier activating enzyme 6
	10.12	FHL2	NM_001450	Four and a half LIM domains 2
	9	QKI	AL031781	Quaking homolog, KH domain RNA binding (mouse)
	8.91	XRN1	AY137776	5-3 exoribonuclease 1
	8.68	PSMD6	AK054730	Proteasome (prosome, macropain) 26S subunit, non-ATPase, 6
	8.43	SAV1	BF983202	Salvador homolog 1 (Drosophila)
	8.15	WWP2	BC000108	WW domain containing E3 ubiquitin protein ligase 2
	7.51	RNF19B	AL031602	Ring finger protein 19B
	7.39	SOCS4	NM_080867	Suppressor of cytokine signaling 4
	6.27	FBX011	NM_025133	F-box protein 11
	5.57	FBXW7	BE551877	F-box and WD repeat domain containing 7
	5.39	G2E3	AA642341	G2/M-phase specific E3 ubiquitin ligase
	7.72	NUP160	AK026236	Nucleoporin 160 kDa
	7.66	AP1S3	AI474433	Adaptor-related protein complex 1, sigma 3 subunit
	7.59	ZNF460	X78931	Zinc finger protein 460
	6.37	AHCYL1	AK025372	S-adenosylhomocysteine hydrolase-like 2
	7.48	FKBP15	AW340329	FK506 binding protein 15, 133 kDa
Energy sensing/protein kinase inhibition	74.11	PTEN	AI917390	PTEN (genecard)
	25.33	FNIP1	BF677986	Folliculin interacting protein 1
	23.67	FLCN	AA992036	Folliculin
	19.72	BRAF	AW184034	v-raf murine sarcoma viral oncogene homolog B1
	19.19	NDUFA10	BC031332	NADH dehydrogenase (ubiquinone) 1 alpha subcomplex, 10, 42 kDa
	18.96	SMEK2	BC032531	SMEK homolog 2, suppressor of mekl (Dictyostelium)
	16.38	PHLDA3	NM_012396	Pleckstrin homology-like domain, family A, member 3
	11.62	PRKAB2	NM_005399	Protein kinase, AMP-activated, beta 2 non-catalytic subunit
Solute transporters	18.12	SLC12A6	NM_005135	Solute carrier family 12 (potassium/chloride transporters), member 6
	9.35	SLC16A6	NM_004694	Solute carrier family 16, member 6 (monocarboxylic acid transporter 7)
	7.34	SLC30A1	AI553933	Solute carrier family 30 (zinc transporter), member 1
	6.42	SLC41A2	AL136828	Solute carrier family 41, member 2
	5.96	SLC6A8	AW276522	Solute carrier family 6 (neurotransmitter transporter, creatine), member 8
	5.87	SLC6A6	BC006252	Solute carrier family 6 (neurotransmitter transporter, taurine), member 6

### Inhibitor panel screening

Next, a panel of inhibitors was studied for the ability to modulate OXY activity in the context of combination assays (Table 2). A role for mTOR signaling was highlighted by the ability of the mTOR inhibitor rapamycin to significantly impair OXY activity. Similarly, the ability of the AMPK activator AICAR to inhibit OXY activity implicates this sensor as contributing toward activity. The ability of inhibitors of transcription (actinomcyin D), translation (cyclohexamide), and protein degradation (MG132) to moderate OXY activity suggests that cytotoxicity is reliant on de novo transcription, translation, and protein turnover. A novel observation involved the reciprocal effects of the antioxidant *N*-acetyl-l-cysteine (L-NAC) and the glutathione biosynthesis inhibitor butathione sulfoxamine (BSO). Pretreatment with L-NAC afforded protection from the effects of OXY whereas BSO significantly enhanced activity. These results implicate glutathione metabolism perhaps also ROS as central to OXY effects. It should also be noted that the ability of the “kinase inhibitor” AG490 to inhibit OXY activity might actually derive from its antioxidant properties 21. The consistent ability of inhibitors of calcium-mediated signaling (BAPTA [(1,2-bis(o-aminophenoxy)ethane-*N,N,N′,N′*-tetraacetic acid)]/EGTA [ethylene glycol tetraacetic acid], cyclosporine, and calmidazolium) to inhibit OXY activity suggests a role for this pathway in activity. A further promising trend involved the ability of several ion channel modulators to inhibit OXY activity. The following were shown to significantly reduce OXY activity: ouabain (Na^+^/K^+^ ATPase inhibitor), 4-aminopyridine (nonselective K^+^ channel blocker), astemizole (H1 antagonist hERG K^+^ blocker), clotrimazole (Ik1 channel inhibitor), NS1619 (large conductance K^+^ channel BK_Ca_ inhibitor), NS309 (activator of small conductance K_ca_ channels), and TRAM34 (intermediate conductance Ca^2+^ activated K^+^ channel inhibitor). These observations broadly implicate plasma membrane potassium, sodium, and calcium transport as important to activity. A considerable number of other compounds including metabolic inhibitors, kinase inhibitors, and additional ion channel modulators were also studied but failed to elicit a response (see Table S2).

**Table:** Modulation of OXY activity in vitro by select agents

Agent	Primary function	Fold change in OXY activity
Rapamycin	mTOR inhibitor	−40
AICAR	AMPK activator	−42.9
LY294002	PI3K inhibitor	−33.3
Actinomycin D	Inhibitor of transcription	−60
MG132	Proteasome inhibitor	−50
Cyclohexamide	Protein synthesis inhibitor	−3
L-NAC	Antioxidant	−4.31
BSO	GSH inhibitor	4500
AG490	Antioxidant and kinase inhibitor	−107.1
BAPTA/EGTA	Ca^2+^ chelator	−32
Cyclosporin	MPT and calcineurin inhibitor	−15
Calmidazolium	Calmodulin inhibitor	−35.2
NH4Cl	Lysosome neutralizer	−60
Cyclosporin	MPT and calcineurin inhibitor	−15
Ouabain	Na^+^/K^+^ ATPase inhibitor	5.8
4-Aminopyridine	Nonselective K^+^ channel blocker	−90.9
Astemizole	H1 antagonist hERG K^+^ blocker	−6
Clotrimazole	Ik1 channel inhibitor	−120
NS1619	Large conductance K^+^ channel BKCa inhibitor	−4.4
NS309	Activator of small conductance Kca^+^ channels	−12
TRAM34	Intermediate conductance Ca^2+^ act. K^+^ channel inhibitor	−33.3
Poly I:C	IAP enhancer	−3.2

OXY, oxyphenisatin acetate; mTOR, mammalian target of rapamycin; AMPK, AMP-activated protein kinase; L-NAC, N-acetyl-L-cysteine; BSO, butathione sulfoxamine; MPT, mitochondrial permeability transition; IAP, inhibitor of apoptosis.

### OXY induces autophagy and mitochondrial dysfunction

**Figure 2:**
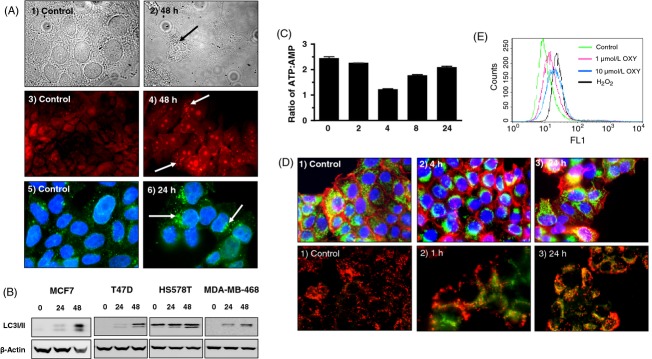
Ten micromoles per liter OXY treatment results in autophagy and mitochondrial dysfunction. (A) Formation of lysosomal structures in MCF7 cells treated for 48 h was determined by compound light microscopy (1,2) and staining with 1 μg/mL acridine orange (3,4). Autophagosome formation was determined 24 and 48 h following treatment by detection of LC3B by immunocytochemistry (5,6). Arrows indicate lysosomal structures. Data are representative of at least three experiments. (B) Time course western blot analysis of LC3I/II expression in a panel of cell lines following treatment. (C) ATP and AMP levels were determined by HPLC as described in Methods in MCF7 cells following treatment for the times indicated. The assay was performed at least twice and the data pooled. Results are expressed as a ratio of ATP:AMP. (D) Upper panels – MCF7 cells treated for 4 or 24 h, fixed, and stained with anti-MTCO2 (green) to detect mitochondrial localization as well as counterstained with DAPI (blue, nucleus) and rhodamine phalloidin (red, actin cytoskeleton). Lower panels – MCF7 cells treated for 1 or 24 h and stained with JC-1 for 30 min. Green (depolarized mitochondria) and red (healthy mitochondria) fluorescence were captured at the same intensity throughout the experiment. (E) FACS analysis of MCF7 cells loaded with the ROS sensor CM-H_2_-DCFDA and treated with OXY for 1 h. Cells in the figure were visualized at 63× original magnification. OXY, oxyphenisatin acetate; ROS, reactive oxygen species.

**Figure 3:**
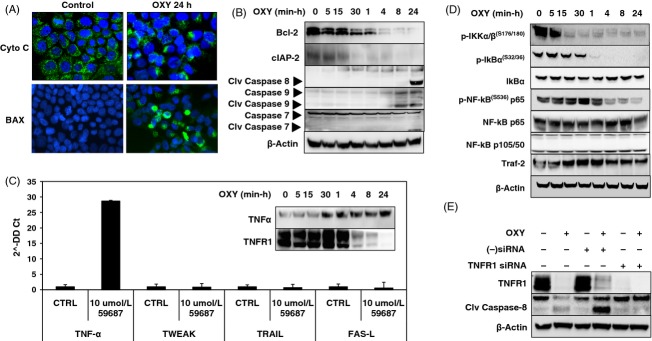
Prolonged exposure to 10 μmol/L OXY promotes both intrinsic and extrinsic apoptosis. (A) MCF7 cells were incubated with OXY for 24 h and stained for expression/localization of cytochrome c and Bax. Cells visualized at 63× original magnification. (B) Western blotting of time course lysates from MCF7 cells treated with OXY for apoptosis-related events. (C) qRT-PCR of changes in mRNA expression for TNF-α, TWEAK, TRAIL, and FasL following 24 h treatment. Inset; western blotting time course of TNF-α and TNFR1 expression in MCF7 cells. (D) Western blotting of NF-κB signaling components and their respective phosphostates following exposure to OXY. (E) Western blotting of cleaved caspase 8 in scrambled and TNFR1 siRNA-transfected MCF7 cells treated with OXY for 24 h. OXY, oxyphenisatin acetate; qRT-PCR, quantitative real time polymerase chain reaction.

**Figure 4:**
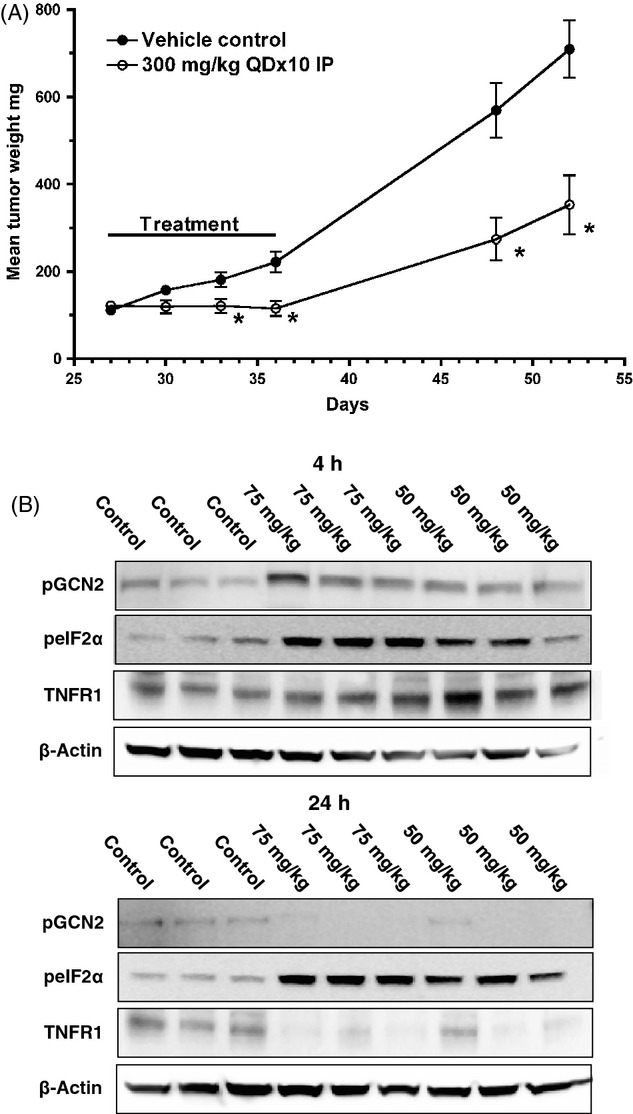
OXY reduces tumor growth in an MCF7 xenograft animal model and induces phosphorylation of GCN2 and eIF2α. (A) Effect of IP administration of OXY 300 mg/kg QD × 10 on the growth of subcutaneously implanted MCF7 xenografts. All treated group tumors were statistically significantly smaller than the control group at each time point from 33 days to termination of the experiment at day 52 (*P* ≤ 0.05). (B) Athymic nude mice harboring MCF7 xenografts were treated with OXY at 50 or 75 mg/kg IV for the indicated times (three mice per time point). Tumor lysate was then prepared and immunoblotted for expression of pGCN2, peIF2α, and TNFR1. OXY, oxyphenisatin acetate; eIF2α, eukaryotic translation initiation factor 2α; IP, intraperitoneal.

As OXY was shown to inhibit translation and microarray analysis implicated active autophagy, further evidence for induction of autophagy was sought. OXY treatment resulted in formation of punctate cytoplasmic structures without altering nuclear or membrane integrity, a characteristic of autophagy induction 22 (Fig. 2A, 1,2). Confirmation that these punctate structures were the acidic compartments typically associated with autophagy 22 is shown by the greater intensity and punctate localization of acridine orange, a compound that accumulates in acidic structures and upon protonation produces a bright red fluorescence (Fig. 2A, 3,4). Another hallmark of autophagy involves relocalization of diffuse cytosolic LC3 to more punctate staining with the formation of autophagosomes. During autophagy, LC3 is cleaved to LC3-I and converted to LC3-II through lipidation to become associated with autophagosomes 22. Treatment with OXY for 24 h resulted in relocalization of LC3 (Fig. 2A, 5,6) and protein cleavage as confirmed by western blotting in MCF7 and several other cell lines (Fig. 2B).

On the basis of previously noted AMPK activation we investigated whether OXY treatment was associated with altered levels of AMP and ATP. Figure 2C shows that the ratio of ATP:AMP in MCF7 cells decreased from 2–4 h following treatment and was gradually restored by 24 h, indicating that OXY causes a transient loss of ATP. This time course of ATP depletion and restoration correlates with the time course of activity of AMPK typically reported in the literature following nutrient deprivation 23 and also with the effect of OXY on AMPK. This movement is indicative of transient energy crisis 24. The ability of OXY to transiently reduce ATP levels prompted an investigation into the effects of OXY on mitochondria. MCF7 cells treated with OXY for 4 or 24 h were stained with markers of mitochondria (MTCO2), actin cytoskeleton (rhodamine phalloidin), or nuclei (DAPI). Analysis by immunofluorescence microscopy (Fig. 2D, upper panels) showed in untreated cells, mitochondria were distributed throughout the cytoplasm, whereas at 4 h of treatment when levels of ATP were low, mitochondria formed distinct unipolar clusters at the nuclear membrane. By 24 h when the ATP levels were restored, mitochondria were also restored to their pretreatment localization within the cytoplasm. Further confirmation that mitochondrial dysfunction contributes to OXY activity is provided by microscopic analysis using the fluorescent indicator JC-1 which aggregates in healthy mitochondria and emits a red fluorescence. A decline in mitochondrial transmembrane potential (Δψm) results in the release of JC-1 from the mitochondria, which leads to its disaggregation into monomers and the appearance of green fluorescence. As shown in Figure 2D, lower panels, OXY induced mitochondrial depolarization after only 1 h of treatment. Lastly, FACS (fluorescence-activated cell sorting) experiments with the ROS sensor CM-H_2_-DCFDA (Fig. 2E) confirmed that treatment was associated with modest ROS generation, which supports inferences from inhibitor screening studies (Table 2) showing that ROS and/or glutathione metabolism is important to the effects of OXY.

### OXY promotes autocrine TNFα-mediated apoptosis

The mechanism of OXY-mediated cell death was then investigated in greater depth. Translocation of Bax to the mitochondria and subsequent release of cytochrome c are hallmarks of mitochondrial-mediated apoptosis 25,26. Immunofluorescence of MCF7 cells with antibodies against these proteins (Fig. 3A) confirmed that OXY induced an upregulation and localization of Bax as well as promoted the release of cytochrome c. Subsequent western blotting of lysates prepared from OXY-treated MCF7 cells revealed that levels of the antiapoptotic proteins Bcl-2 and cIAP-2 (inhibitor of apoptosis-2) began to decrease after only 30 min of treatment, followed by cleavage of pro-apoptotic caspases 8, 7, and 9 (Fig. 3B). Together these results suggest that OXY evokes an apoptotic response involving mitochondrial events.

Cleavage of caspase 8 is indicative of extrinsic (receptor-mediated) apoptosis 27. In order to identify the ligand(s) responsible for this effect, qRT-PCR was performed on mRNA from MCF7 cells treated with OXY at 10 μmol/L for 24 h (Fig. 3C). Results demonstrated that out of four ligands examined (TNF-α, TWEAK, TRAIL, FasL), only TNF-α mRNA was enhanced following treatment. This trend was mirrored at the protein level and accompanied by a rapid degradation of the TNF-α receptor (TNFR1) (Fig. 3C, inset), which is indicative of receptor ligation and signaling 28,29. Enhanced TNF-α (mRNA and protein), along with degradation of TNFR1 was also noted in another sensitive line T47D but not for resistant MDA-MB-231 cells (results not shown). It is recognized that NF-κB survival signaling can inhibit the TNF-α-mediated death response 30–33. As shown in Figure 3D, treatment of MCF7 cells with OXY resulted in the inhibition of the IKK/IκBα/NF-κB signaling pathway. These events will likely enhance the effect of any autocrine TNF-α production. Lastly, siRNA knockdown of TNFR1 confirmed that receptor ligation was likely the event responsible for caspase 8 cleavage (Fig. 3E). Overall, these results suggest that OXY treatment leads to death receptor signaling driven by autocrine TNF-α production.

### OXY is active in vivo where it induces phosphorylation of GCN2 and eIF2α accompanied by degradation of TNFR1

Next, in order to reaffirm the potential of OXY-like molecules as clinical candidates, the agent was assessed for in vivo antitumor activity in an MCF7 xenograft animal model. Toxicity studies demonstrated that mice tolerated IP administration of OXY at 300 mg/kg once daily or 200 mg/kg twice daily. Administration of OXY at 300 mg/kg IP once daily for 10 days resulted in significantly smaller tumors from day 33 to day 52 (*P* < 0.05) (Fig. 4A). Consistent with in vitro data, tumors did not grow during the treatment period. However, following cessation of treatment, tumor growth rate increased albeit at a slower rate than the vehicle control group. Two mice from the treatment group were sacrificed, not due to toxicity from OXY, as this dose has been administered to mice without incident and with nominal weight loss (4.8%), but to estradiol toxicity, a problem with chronic estrogen supplementation required for tumor growth in MCF7 xenografts 34. Overall, these results mirrored in vivo observations made for structurally related molecules 12.

Finally, in order to provide evidence that molecular observations in vitro were also relevant to in vivo activity, tumors were collected at 4 and 24 h following a single IV dose of 50 or 75 mg/kg OXY. As shown in Figure 4B, GCN2 phosphorylation increased at both doses in tumors 4 h post treatment compared with vehicle control, but by 24 h the effect had disappeared, a pattern consistent with the transient upregulation seen in vitro (Fig. 1E). Furthermore, phosphorylation of eIF2α was significantly elevated at both time points and doses, also consistent with the sustained response observed in vitro (Fig. 1E). Interestingly, also similar to in vitro results, TNFR1 levels had almost disappeared 24 h after administration (Fig. 4B). While no pharmacokinetic data for oxyphenisatin in mice has been published, it is apparent that a biologically active dose can be achieved at 50 mg/kg (1.2 mmol/L assuming 2 mL blood volume) as determined by these pharmacodynamic end points. These data confirm similar results to those obtained in vitro and suggest, on the basis of rapid and sustained induction, that peIF2α increases represent the most attractive candidate for an OXY response biomarker.

## Discussion

Enhanced metabolic demands make cancer cells especially vulnerable to compounds targeting nutrient uptake or utilization. Data generated in this study suggest that OXY is capable of inducing a rapid multimodal starvation response in cancer cells.

eIF2α is a convergence point for several stress response pathways. Phosphorylation by upstream kinases such as GCN2, PERK, and PKR results in inhibition of translation. Phosphorylation of GCN2 occurs as a result of accumulation of uncharged tRNA 35,36, whereas PERK is activated when reduced ATP concentrations inhibit sarcoplasmic/ER Ca^2+^-ATPase (SERCA) pumps 37. Activation of both GCN2 and PERK was one of the earliest events noted following treatment, supporting a decline in intracellular tRNA accompanied by ER/ATP stress. Similarly, AMPK plays an important role in cellular homeostasis by sensing the ATP/AMP ratio 38. AMPK and other signals are then integrated by mTOR, a master controller of metabolism, growth, and redox potential 39. Activation of AMPK accompanied by a decline in phosphorylation of two mTOR substrates (p70S6K and 4E-BP1) confirms that these regulators were perturbed following treatment. The importance of AMPK signaling was further strengthened by data showing that preactivation of this system with AICAR moderates the toxic effects of OXY. Importantly, a transient decline in ATP was noted and provides a basis activation of both AMPK and PERK.

These data mirror those reported for related molecules 12 showing phosphorylation of GCN2, EIF2α, AMPK and reduced phosphorylation of the mTOR substrates p70S6K and 4E-BP1. Intriguingly, the authors also note (using a metabolomics platform) that by 4.5 h, intracellular levels of several amino acids (including glycine, threonine, and histidine) decline significantly following treatment 12. Taken together, these data implicate plasma membrane solute flux as a possible originating event. We also speculate that the ability of the sulfhydryl-containing antioxidant L-NAC to ablate OXY activity, and the glutathione biosynthesis inhibitor BSO to enhance activity, may originate from the reduced glutathione tripeptide biosynthesis as a consequence of amino acid depletion.

The case for a cell starvation response was further strengthened by results demonstrating induction of macroautophagy, a catabolic process that involves degradation of dysfunctional proteins and organelles in an effort to recycle amino acids and fatty acids for energy utilization and cell survival 40–42. Autophagy is classically induced in response to the activation of AMPK and subsequent inhibition of mTOR 43. Here, observations such as enhanced acridine orange staining and LC3 cleavage confirmed the existence of autophagy. Likewise, perturbations in energy metabolism and autophagy should be accompanied by mitochondrial dysfunction. Results confirmed that OXY treatment was indeed associated with both unipolar clustering and depolarization of mitochondria.

Continuous exposure to OXY appeared to trigger both intrinsic and extrinsic modes of apoptosis. The intrinsic pathway is characterized by the activation of effector caspases 3 and 7 that occur upon release of cytochrome c following permeabilization of the mitochondria 44,45. Extrinsic receptor mediation is associated with cleavage of caspase 8 26. All of the above events were noted following 24 h exposure to OXY. Previous work noted that the cytotoxicity of related molecules can be alleviated by pretreatment with the caspase inhibitor z-VAD-fmk 12.

The ligand(s) mediating extrinsic apoptosis induction were of significant interest. After exploring the expression of several death receptor/ligand combinations it was shown that expression of TNF-α was elevated following treatment. TNF receptor ligation is associated with endocytosis, release of the death complex, and subsequent degradation of the receptor 28,46. OXY treatment resulted in a decrease in the expression of the TNFR1 prior to activation of caspase cleavage, implicating this signaling cascade as central to apoptosis induction. Final confirmation for a role for TNF-α came from siRNA experiments showing that TNFR1 signaling was essential for caspase 8 cleavage. It should be noted that inhibition of protein synthesis is recognized as a classical initiator of autocrine TNF-α mediated apoptosis 47. Numerous cellular processes associated with the inhibition of protein synthesis enhance sensitization to death receptor stimuli. Critically important events are inactivation of the pro-survival NF-κB pathway 33, activation of the AMPK pathway 48, the initiation of the mitochondrial permeability transition (MPT) 49, along with the induction of both autophagy and ROS 22,30,50,51. In this regard, autocrine TNF-α-mediated cell death is a logical outcome following OXY treatment.

In summary, these data suggest that OXY treatment is associated with a rapid decline in intracellular metabolites leading to simultaneous activation of nutrient sensing pathways. Sustained activation leads to apoptosis induction, mediated to some extent by autocrine TNF-α. Interestingly, ER+ cell lines MCF7 and T47D showed the greatest OXY sensitivity and were the only cell lines that showed increased transcription of TNF-α in response to OXY. Recent studies have shown that estrogen can play a role in regulation of transcription of TNF-α 52. Unpublished results from our laboratory suggest that OXY treatment reduced the level of ERα, known to be downregulated by TNF-α 53. This decrease in the expression of ERα may cause cells that are dependent on estrogen for growth and survival to become increasingly susceptible to treatments that induce a TNF-α response, such as OXY, thus explaining why the ER^+^ MCF7 and T47D cells show a greater sensitivity to OXY compared to ER^−^ cell lines MDA-MB-468 and HS578T. However, the presence of the ER is not an absolute requirement for cell death induced by OXY as activation of the intrinsic pathway of apoptosis is sufficient to cause cells to commit to apoptosis 54. Yet, the molecular target responsible for activity remains undefined. Data collected thus far appears to implicate a ubiquitous controller of intracellular solute concentrations as a potential candidate. Pleasingly, the starvation response and growth suppression was also evident in xenograft models and did not appear to be overtly toxic. As a consequence OXY remains an interesting pharmacophore for further investigation. It should also be recognized that the wealth of preexisting data relating to OXY makes the parent molecule itself a compelling development candidate.
