# Supplementary material for: Oxyphenisatin acetate (NSC 59687) triggers a cell starvation response leading to autophagy, mitochondrial dysfunction, and autocrine TNFα-mediated apoptosis
Source: Cancer Med. 2013 Jul 23;2(5):687–700. doi: 10.1002/cam4.107 (PMC3892800; doi:10.1002/cam4.107)
Supplement: Supplementary file 2 [file cam40002-0687-sd2.pdf]

Agent	Primary Activity
3-Methyladenine	Autophagy Inhibitor
A769662	AMPK activator
Amiadorone	Non-selective ion channel blocker
Apamin	Small conductance KCa2+ channel blocker
Brefeldin	Protein transport inhibitor
CGP-37157	Na+/Ca+ Exchange Inhibitor
chloroquin	Lysosome neutralizer
Compound C	AMPK Inhibitor
Compound C	AMPK Inhibitor
CPG 57380	MNK1 Inhibitor
Cromakalim	K)ATP) channel opener
DCEBIO	Activates apical Cl- conductance
Deoxyglucose	Inhibitor of glycolysis
DET	SOD1 Inhibitor
Forskolin	Adenylate cyclase activator
Genestein	Kinase Inhibitor
Glibenclamide	ATP-dependent K^+ channel and CFTR Cl^- channel blocker
GSK 650934	SGK1 Inhibitor
GSK3B inhibitor XII	GSK3B Inhibitor
GW5074	RAF1 Inhibitor
Iberiotoxin	Large conductance K+ channel BKCa inhibitor
Iodotubercidin	Adenosine kinase inhibitor
KT5823	PKG Inhibitor
Linoperdine	KCNQ K+ channel blocker
Manumycin A	Ras Inhibitor
metformin	Nucleoside transport inhibitor
NFATi	NFAT Inhibitor
NFKBi	NFKB Inhibitor
Oligomycin	ETC Complex V Inhibitor
Paxilline	Maxi K+ Channel Blocker
PD98059	MEK Inhibitor
Perhexilline	CPT-1 Inhibitor
Phloretin	PKC Inhibitor
Pinacidil	K+ channel activator
Ro31-8220	PKC Inhibitor
Rotenone	ETC Complex I inhibitor
Rp-cAMPs	cAMP Kinase Inhibitor
SB203580	p38 MAPK Inhibitor
SL0101-1	RSK1 Inhibitor
SM-122	IAP Antagonist
SMAC-N7 peptide	IAP antagonist
Sp-cAMPs	cAMP kinase activator
SP600125	JNK Inhibitor

staurosporin	Kinase Inhibitor
Terfenadine	hERG and KATP channel blocker
Tetraethylammonium chloride	Non-selective K+ channel blocker
Wortmannin	PI3K Inhibitor
